# Intrathecal Digoxin Administration During Spinal Anesthesia: A Case Report Outlining Recovery and Rehabilitation

**DOI:** 10.7759/cureus.65376

**Published:** 2024-07-25

**Authors:** Nicholas R Hooper, Minh Andy Doan, Derick Davis, James Morgan

**Affiliations:** 1 Department of Physical Medicine and Rehabilitation, Emory University, Atlanta, USA; 2 Department of Spinal Cord Injury, Shepherd Center, Atlanta, USA

**Keywords:** musculoskeletal rehabilitation, obstetric anesthesia, acute encephalitis, spinal cord injury, physical medicine and rehabilitation

## Abstract

This case report highlights the rehabilitation outcomes of a 24-year-old female who received inadvertent intrathecal digoxin during a routine cesarean section, leading to encephalomeningitis, vestibulocochlear neuritis, and incomplete paraplegia. Despite initial neurological deficits, the patient demonstrated significant improvement in both cognition and functional mobility during a one-month inpatient rehabilitation program, ultimately achieving ambulation with assistive devices. This case underscores the potential for rehabilitation of neurological sequela following accidental intrathecal digoxin administration.

## Introduction

The systemic effects of digoxin toxicity are well-documented but reports of neuraxial administration remain scarce [1-3]. Only a few case reports have previously examined the effects of accidental intrathecal digoxin, with symptoms including encephalopathy, paraplegia, and brain death. Most previous reports describe spontaneous resolution of neurologic sequelae within 2-7 days with minimal intervention [1]. Although permanent impairments have been reported, rehabilitation outcomes in such cases have not been extensively explored and to our knowledge, there is no existing literature describing functional progress. This case report aims to fill this gap by describing the rehabilitation outcomes of a patient following inadvertent intrathecal digoxin administration.

## Case presentation

A 24-year-old female underwent a planned cesarean section, during which she received intrathecal digoxin instead of bupivacaine. During the C-section, she underwent spinal anesthesia, and although no surgical complications were initially reported, post-operatively it was discovered that 375 mcg of intrathecal digoxin was accidentally administered instead of bupivacaine for the anesthetic block. Subsequently, she experienced respiratory distress requiring intubation, encephalopathy, and bilateral hearing loss. Digoxin-specific antibody (DIGIFab) was administered approximately four hours after accidental digoxin administration, followed by 400mg of DigiBind (an alternative digoxin-specific antibody) and high-dose steroids hours later (1g solumedrol followed by five-day pulse of 1g methylprednisolone).

**Figure 1:**
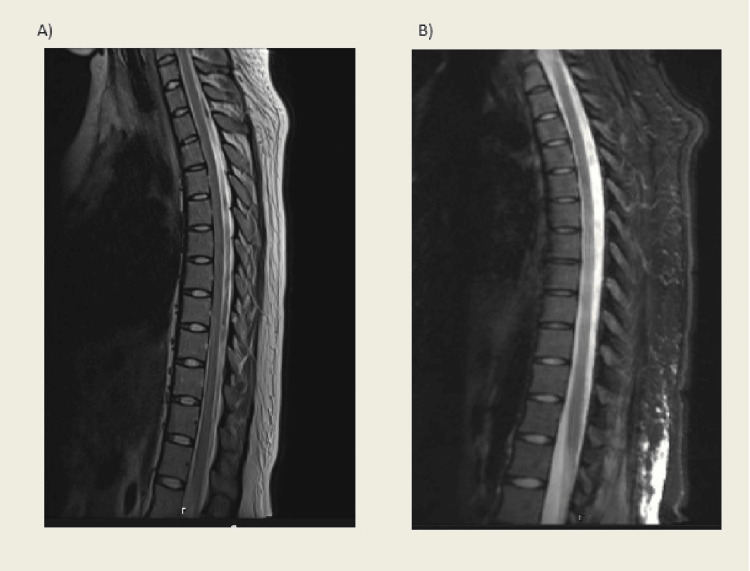
T2 weighted MRI of thoracic spine without spinal cord abnormality. A represents MRI completed on post-op day #1 and B represents the MRI completed on post-op day #21. Both are normal without any obvious etiology of paralysis.

Initial work-ups including CT and CTA of head/neck, as well as MRI brain and thoracic spine, were all unremarkable (see Figure 1A). Repeat MRI on post-op day 21 did not reveal any abnormalities (Figure 1B). A lumbar puncture demonstrated elevated opening pressure of 33 cm and CSF studies were notable for elevated WBC to 4,000s with elevated protein and glucose levels, consistent with meningitis. Vancomycin and piperacillin/tazobactam were started for meningitis and a lumbar drain was placed due to elevated opening pressures.

**Figure 2:**
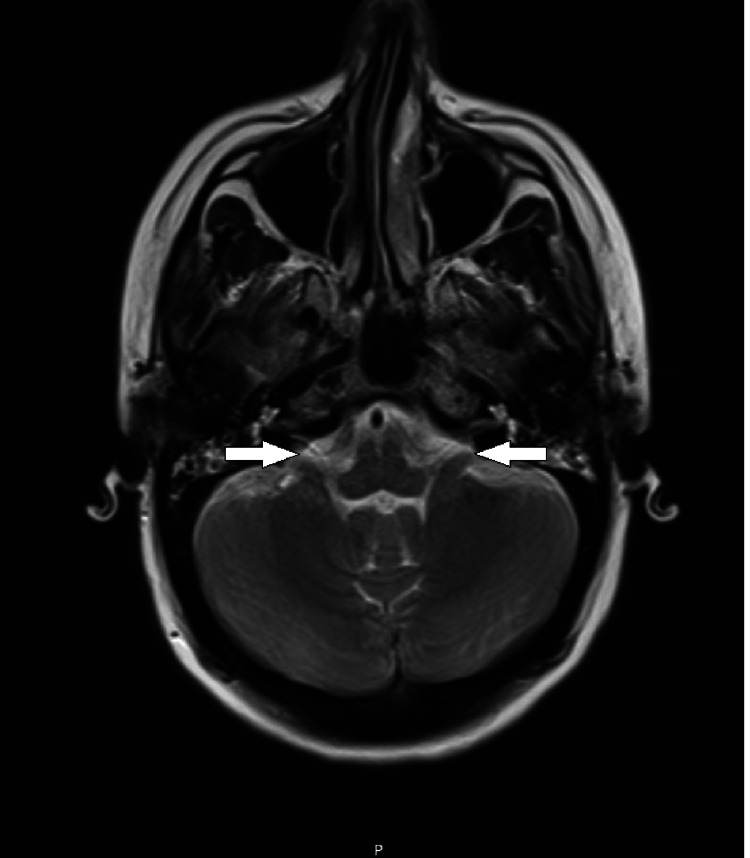
T2 weighted MRI of brain completed on post-op day #12. Imaging with enhancement of bilateral vestibular cochlear nerves consistent with encephalomeningitis. Arrows detail enhancements bilaterally.

On post-op day #1, the patient's trachea was successfully extubated and she was found to have significant bilateral lower extremity weakness with encephalopathy including impairments in both memory and executive function. Repeat MRI brain on post-op day #2 demonstrated diffusion restriction and an abnormal non-enhancing T2/FLAIR signal of the bilateral amygdala and hippocampus, as well as an abnormal T2/FLAIR signal throughout bilateral insula, frontal opercula, cingulate gyri, and medial thalami, suggestive of meningeal and parenchymal inflammation. CSF pressures stabilized and the lumbar drain was successfully removed on post-op day #9. However, she had developed complete hearing loss thought to be ototoxic in nature. Another MRI brain completed on post-op day #12 revealed new onset abnormal enhancement of bilateral vestibular cochlear nerves consistent with encephalomeningitis (see Figure 2). Due to concern for inflammatory etiology, she completed another five-day course of IV methylprednisolone and then was transitioned to an extended taper of oral prednisone (60mg daily and taper 10mg every two weeks). Once medically stable, she transferred to an inpatient rehabilitation (IPR) hospital for specialty medical management and interdisciplinary rehabilitation to address deficits related to acquired brain injury and spinal cord injury.

The patient presented to inpatient rehab 19 days following her initial operation with the initial neurologic exam demonstrating persistent bilateral hearing loss, visual field impairments, cognitive deficits including short-term and working memory impairment, and incomplete paraplegia. Initial International Standards for Neurological Classification of SCI (ISNSCI) exam demonstrated T5 American Spinal Injury Association (ASIA) Impairment Scale (AIS) C. Table 1 shows lower extremity motor scores both on admission and discharge. There were notable flaccid LE weakness, hyporeflexia, and negative Babinski’s reflex. The patient had intact deep anal pressure, negative bulbocavernosus reflex, and absent voluntary anal contraction. Repeat MRI thoracic and lumbar spine at the time of admission to inpatient rehab again showed no spinal cord abnormalities (Figure 1B).

**Table 1:** Lower extremity motor scores on admission and discharge neurologic exam. EHL: Extensor Hallucis Longus

	Admission	Discharge
Hip Flexors L2	1/5	1/5
Knee Extensors L3	0/5	4/5
Ankle Dorsiflexors L4	1/5	4/5
EHL L5	0/5	3/5
Ankle Plantarflexors S1	1/5	5/5

On admission, she was noted to minA to modA for many activities of daily living (ADLs), including lower body dressing, bathing, and transfers. She was also noted to be supervised for wheelchair mobility. Cognitively she required ModA to MaxA for comprehension, problem-solving, and memory. Overall, she scored 46 on the functional independent measure (FIM) motor subscale and 22 on the FIM cognitive subscale on admission. Table 2 shows FIM scores on admission and discharge.

**Table 2:** Functional independence measure (FIM) scores on admission and discharge. FIM scores: Graded as: 7- complete independence, 6- modified independence, 5- supervision, 4- minimal assistance (MinA), 3- moderate assistance (ModA), 2- maximal assistance (MaxA), 1- total assistance (TotA), 0- unable to complete.

	Admission	Discharge
Self Care		
a. Eating	6	6
b. Grooming	6	6
c. Bathing	3	6
d. Dressing- Upper	6	7
e. Dressing- Lower	3	7
f. Toileting	1	6
Sphincter Control		
g. Bladder Management	2	7
h. Bowel Management	2	7
Transfers		
i. Bed, Chair, Wheelchair	4	5
j. Toilet	4	5
k. Tub, shower	4	5
Locomotion		
l. Walk/Wheelchair	5C	5C
m. Stairs	0	0
Motor Total:	46	72
Communication		
n. Comprehension	4	7
o. expression	7	7
Social Cognition		
p. Social interaction	6	7
q. Problem solving	3	5
r. Memory	2	3
Cognitive Total:	22	29

Throughout the hospital course, the patient made significant improvements in neurologic recovery. She had emerging bowel and bladder continence with return of bowel sensation a month following her initial operation. However, the patient did not regain voluntary anal contraction and required daily rectal clears for bowel management for the duration of her inpatient stay. Neuro-ophthalmology exam four weeks following inpatient rehabilitation admission demonstrated right cranial nerve 4, superior oblique palsy managed with a brock string, and prism therapy. On discharge, the patient noted subjective improvements in her vision, but no formal testing was repeated. She also had gradual improvement in hearing loss, scoring within normal limits on auditory screening examination performed via the speech language pathologist prior to discharge, with subjective impairments in background noise discrimination. On discharge, approximately six weeks following admission, the patient had significant strength gains with discharge ISNSCI improved to T10 AIS D, thus gaining five spinal levels (Table 1). The sensory level was found to be T10 possibly confounded by its proximity to recent C-section scar. Of note prior to discharge, she had new onset bilateral ankle clonus and emerging spasticity in her lower extremities. Discharge neuropsychiatric examination demonstrated continued cognitive deficits with exceptionally low ratings in visuospatial/constructional and delayed memory categories but average-exceptional ratings in other cognitive domains.

On discharge, her functional status greatly improved. She scored a 72 on the FIM motor subscale and 29 on the FIM cognitive subscale on admission (Table 2). She required supervision to modified independence for most ADLs. Although still rated as supervision for wheelchair mobility, she started to ambulate by the end of her acute inpatient stay. She was able to ambulate multiple bouts of 50 feet with MinA and a rolling walker and bilateral malleolocs. Her timed velocity with therapy was .19 m/s.

Following another four weeks of outpatient therapy, she was noted to be ambulating with modified independence using a rollator and bilateral AO braces. Her gait velocity had increased to .55 m/s. In addition, she was noted to be able to ascend/descend four stairs with MinA and bilateral rail support. Her quadriceps, anterior and posterior tibialis, and toe flexor strength all improved from 3/5 to 5/5 on manual muscle testing. 

## Discussion

This case contributes to the limited literature on both symptomatology and functional outcomes following intrathecal digoxin administration. A literature search yielded only 10 published cases of inadvertent digoxin administration into the neuraxis during spinal anesthesia and to our knowledge, this is the first case report to examine a patient’s rehabilitation progress after such an incident. All previously reported cases describe some level of motor and sensory changes resulting in either temporary or permanent paraplegia or quadriplegia [1]. Other consistently reported symptoms include seizures [2], meningitis or encephalopathy [1,3], and elevated opening pressure with lumbar puncture [2,3]. Five of these 10 previous case reports describe peripartum patients who developed symptoms after accidental intrathecal digoxin administration during the cesarian section. Additionally, and importantly, pregnancy appears to be a risk factor for developing significant morbidity [4]. In addition to some level of motor and sensory loss, the patients developed central nervous system symptoms including agitation, seizures, and encephalopathy/encephalitis. Overall, 60% (3 out of 5) of these peripartum cases describe significant permanent neurologic sequela, one of which, unfortunately, resulted in death.

While the patient discussed in this current case developed the more commonly reported sequelae of encephalitis and paraplegia, she uniquely also developed vestibulocochlear neuritis with complete hearing loss, a previously unreported complication. In addition, abnormal bilateral vestibular cochlear nerve enhancement was seen on MRI. While prior reports do not always publish or discuss imaging findings, it appears the available imaging reports describe inconsistent findings. Abdallah et al. described CT/MRI of the CNS displayed findings consistent with ischemic damage [3], while others showed edematous central changes [1,2], or no acute abnormalities at all [5,6]. Regarding our patient, acute MRI findings at the outside hospital revealed abnormal changes in multiple areas of the brain including bilateral amygdala, hippocampus, bilateral insula, frontal opercula, cingulate gyri, medial thalami, and bilateral vestibular cochlear nerves. However, the MRI thoracic and lumbar spine showed no evidence of spinal cord abnormality to explain the central etiology of paraplegia.

Digoxin is a cardiac glycoside that functions primarily to increase contractility of the heart by way of blocking the Na+/K+ ATPase in myocardial cells [7,8]. Specifically, digoxin binds the alpha subunit in cardiac cells, increasing intracellular sodium which allows greater accumulation of calcium ions in the sarcoplasmic reticulum, increasing force during cardiac contraction.

While the adverse effects of systemic digoxin (toxic dosing at or above a serum level of 2.0ng/ml^8^) are well known to include nausea, vomiting, vision problems (xanthopsia), and anorexia, the effects of intrathecal administration are not clearly understood. Neuraxial administration in rabbits via an L3-4 interlaminar approach was found to reliably cause transient motor and sensory loss with return of function after 8-9 minutes (digoxin half-life is 1.5-2 days in humans [9]). However, these findings were dose dependent with larger amounts causing a more severe spinal block followed by the demise of the animal through cardiopulmonary arrest [10]. The mechanism behind such predictable attenuation of motor and sensory function, however, is unclear.

Authors of other similar reports on inadvertent intrathecal digoxin injections postulate that the drug’s ability to bind various alpha subunit isoforms (α1, α 2, α3) on the Na+/K+ ATPase enzyme within neuronal membranes leads to central nervous system edema from accumulation of intracellular sodium [1,11]. Presumably, this edema results in devastating neurologic damage with subsequent paralysis and hypoesthesia. Bagherpour et al. postulated digoxin binding to sodium pumps may result in neural conduction loss [6]. Lastly, Garcia et al. hypothesized that the drug or excipients therein could cause vasoconstriction leading to ischemic damage to neuronal tissue [12]. Such an underlying mechanism would explain prior cases wherein patients seemingly suffered from anoxic brain injuries or spinal cord infarcts.

Treatment following neuroaxial digoxin administration often requires cardiopulmonary and neurologic stabilization (e.g. mechanical ventilation and anti-epileptic treatment in the cases where patients were experiencing seizures). In our case, as well as two previously reported cases, digoxin-specific antibody (DIGIFab) was administered once the etiology of the patients’ symptoms was identified [4]. However, it does not appear that any of these patients, including ours, displayed typical adverse effects seen with systemic digoxin toxicity. Thus, this treatment is of unknown benefit in cases of direct neuroaxial administration of digoxin. Given the occasional findings of edematous central nervous system changes on MRI, steroids have also been used as an adjunct/supportive treatment [4] - as was the case with our patient. Elevated CSF opening pressure with lumbar puncture pressure and CSF lab findings may also suggest meningitis thereby prompting antibiotic/antiviral therapy [3,4]. Again, such a treatment may be of no benefit without other systemic symptoms or signs of infection.

We found no studies specifically evaluating the long-term functional recovery of non-traumatic brain injury and spinal cord injury secondary to digoxin induced aseptic encephalomeningitis. Previous case studies have attributed paraplegia to spinal cord edema secondary to chemical irritation, with greater impairments in the lower extremities due to gravity dependent diffusion of the drug [4]. In the 10 cases of paraplegia secondary to intrathecal administration of digoxin, 50% of patients had permanent neurologic deficits. In our patient's case, there was significant improvement in motor and sensory function during the first 2 months following initial injury with total motor scores improving from 56 to 84.

There have been no prior documented cases of hearing loss secondary to intrathecal digoxin. Other cases of hearing loss secondary to intrathecal drug administration were seen in known ototoxic medications such as vancomycin and methotrexate-cytarabine resulting in severe long term sensorineural hearing loss [13,14]. This patient had subjective recovery of hearing loss at one month likely due to the decreased ototoxic side effect profile of digoxin, although it is unclear the extent of injury and recovery, as no formal audiologic testing was performed other than screening tests.

Cognitive impairments are common after aseptic meningitis and pronounced impairments can be seen in memory, executive function, and concentration. Fortunately, good recovery with little-to-no neuropsychological deficits can be seen at one year [14]. Although our patient showed improvements in memory and executive function, she had persistent cognitive impairments at time of discharge from inpatient rehab.

Morbidity, especially for peripartum patients, following intrathecal digoxin administration can be catastrophic [4,12]. A case published in 2023 by Tabaac et. al reviews the case of a young pregnant patient who accidently received neuraxial digoxin instead of routine epidural bupivacaine, which resulted in death [1]. Indeed, as previously mentioned, such an event gives a 50% chance of significant long-lasting neurologic damage [4]. With the first identifiable case report dating back to 2006 [6], it is glaringly apparent that these preventable injuries continue to occur. Multiple reports suggest the stocking of digoxin and bupivacaine in proximity as a culprit as their vials can often appear similar [1,4,12]. With the severity of patient suffering, the authors hope other safeguards can be put in place to prevent further episodes of accidental digoxin administration in the intrathecal or epidural space. 

## Conclusions

Rehabilitation following inadvertent intrathecal digoxin administration is feasible and can lead to significant improvements in neurological function. Here we report on the rehabilitation and recovery of a 24-year-old female who received inadvertent intrathecal digoxin during a routine cesarean section, leading to encephalomeningitis, vestibulocochlear neuritis, and incomplete paraplegia. She demonstrated significant cognitive, auditory, and functional improvements throughout inpatient rehab. By the time of discharge, she scored within normal limits on auditory screening exams, displayed improved memory, and converted from T5 AIS C incomplete paraplegia to T10 AIS D, paraplegia. Even with these improvements, she still displayed persistent sequelae, including deficits in visuospatial processing, delayed recall memory, and lower extremity sensorimotor impairments. Therefore, she transitioned to a month-long outpatient rehabilitation day program and continued to improve to the point that she was ambulating independently with assistive devices. This case underscores the importance of multidisciplinary rehabilitation in managing patients who sustain complex neurological injuries following accidental intrathecal digoxin administration. Further research is warranted to better understand the mechanisms underlying neurological recovery in these cases and hopefully prevent future occurrences.
